# Polydopamine Interfacial Coating for Stable Tumor-on-a-Chip Models: Application for Pancreatic Ductal Adenocarcinoma

**DOI:** 10.1021/acs.biomac.4c00551

**Published:** 2024-07-31

**Authors:** Soraya Hernández-Hatibi, Pedro Enrique Guerrero, José Manuel García-Aznar, Elena García-Gareta

**Affiliations:** † Multiscale in Mechanical & Biological Engineering Research Group, Aragon Institute of Engineering Research (I3A), School of Engineering and Architecture, University of Zaragoza, 50018 Zaragoza, Aragon, Spain; ‡ Department of Biochemistry and Molecular and Cellular Biology, Faculty of Sciences, University of Zaragoza, 50009 Zaragoza, Aragon, Spain; § Aragon Institute for Health Research (IIS Aragon), Miguel Servet University Hospital, 50009 Zaragoza, Aragon, Spain; ∥ Division of Biomaterials & Tissue Engineering, UCL Eastman Dental Institute, University College London, London WC1E 6BT, U.K.

## Abstract

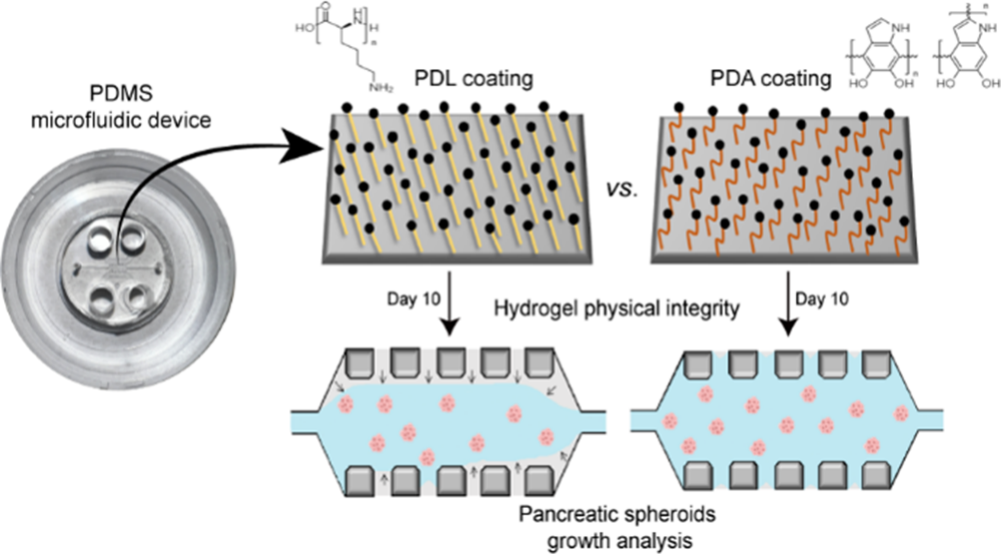

Addressing current challenges in solid tumor research requires advanced in vitro three-dimensional (3D) cellular models that replicate the inherently 3D architecture and microenvironment of tumor tissue, including the extracellular matrix (ECM). However, tumor cells exert mechanical forces that can disrupt the physical integrity of the matrix in long-term 3D culture. Therefore, it is necessary to find the optimal balance between cellular forces and the preservation of matrix integrity. This work proposes using polydopamine (PDA) coating for 3D microfluidic cultures of pancreatic cancer cells to overcome matrix adhesion challenges to sustain representative tumor 3D cultures. Using PDA’s distinctive adhesion and biocompatibility, our model uses type I collagen hydrogels seeded with different pancreatic cancer cell lines, prompting distinct levels of matrix deformation and contraction. Optimizing the PDA coating enhances the adhesion and stability of collagen hydrogels within microfluidic devices, achieving a balance between the disruptive forces of tumor cells on matrix integrity and the maintenance of long-term 3D cultures. The findings reveal how this tension appears to be a critical determinant in spheroid morphology and growth dynamics. Stable and prolonged 3D culture platforms are crucial for understanding solid tumor cell behavior, dynamics, and responses within a controlled microenvironment. This advancement ultimately offers a powerful tool for drug screening, personalized medicine, and wider cancer therapeutics strategies.

## 1 Introduction

Cancer continues to pose a significant challenge to global health and remains a leading cause of mortality.^1^ As a result, intensive research and innovative approaches are necessary to both treat and understand the underlying mechanisms. In the field of cancer research, the significance of the tumor microenvironment (TME) cannot be overstated. The TME is a complex and dynamic environment composed of stromal cells, immune cells, soluble factors, and the extracellular matrix (ECM).^2^ Its chemical and cellular elements, as well as its biomechanical properties have a considerable impact on cancer initiation and development.^3,4^ In recent years the ECM, in particular, has been recognized as a key player in tumor development. The ECM is a complex network of proteins and carbohydrates that provides structural support to tissues and organs.^2^ In the context of tumors, the ECM undergoes significant remodeling, which creates a microenvironment that promotes tumorigenesis and metastasis.^5^ In solid tumors, this ECM has become an important target for study. In particular, in pancreatic ductal adenocarcinoma (PDAC) where up to 90% of the tumor volume could be stroma.^6^

Pancreatic cancer is a significant medical challenge, ranking as the seventh most common cause of cancer-related deaths.^7^ Regrettably, the occurrence of pancreatic cancer has been consistently rising over the past few decades, with little improvement in survival rates over time.^1,8^ Late diagnosis and limited response to conventional treatments, including chemotherapy, surgery, and radiotherapy, are significant factors contributing to lethality associated with pancreatic cancer. The most prevalent variant of this cancer is PDAC, characterized by a nonimmunogenic, immune-suppressive, and therapy-resistant microenvironment. Further complicating advancements in treatment and diagnosis and increasing associated costs.^9^ Therefore, unraveling the biological intricacies of this cancer type assumes critical importance in enhancing patient outcomes and elevating survival rates.

The usual approach to in vitro cancer research relies on using primary cultures or immortalized cell lines created from surgically resected tumors. However, these conventional 2D cell culture methods excessively simplify the mechanical and architectural features of the TME. Moreover, the inherent adaptation of cells to 2D setups remains inadequately understood, resulting in a lack of faithful representation of tumor heterogeneity. Since tumor formation is inherently a three-dimensional (3D) process, the use of 3D culture methodologies is crucial to replicate more realistically in vivo cellular behaviors by allowing manipulation and modulation of the microenvironment. This approach bridges the gap between in vitro and in vivo conditions.^10^ Among the different strategies for 3D culture, microfluidics presents a powerful and versatile tool, enabling the miniaturization of 3D cell culture, while providing an environment for cells to grow within ECM hydrogels.^11^ This restricted 3D setting improves the structural and functional differentiation of cells and better replicates physiological traits.^12^ However, despite the significant biological significance of ECM hydrogels, they also present a distinct challenge to the in vitro culture in microfluidic devices. The growth and attachment of cells to these hydrogel scaffolds result in mechanical forces being applied to the surrounding ECM. While forces play a critical role in various biological processes, prolonged force generation can lead to the gradual contraction of the extracellular matrix scaffold. This causes detachment of the hydrogel, which poses a significant obstacle in developing 3D in vitro models for long-term cultures that are physiologically relevant.^13^ This issue poses a significant challenge in PDAC, in which cell lines exert considerable force on the surrounding ECM due to their high invasiveness and ability to remodel the ECM.^14^ To address this challenge, the study of new methods to better support the ECM and prevent contraction is necessary in order to be able to maintain the structural integrity of the hydrogels and to culture pancreatic cancer cell lines for representative periods of time.

Polydopamine (PDA) is an emerging biopolymer inspired by adhesion proteins found in mussels. These proteins contain 3,4-dihydroxy-l-phenylalanine (DOPA) and lysine amino acids, which led to the hypothesis that the coexistence of catechol (DOPA) and amine (lysine) groups can be crucial for achieving strong adhesion.^15^ PDA is formed by spontaneous oxidative polymerization induced by the pH of dopamine hydrochloride in alkaline solutions (pH > 7.5). Immersing substrates in a dilute aqueous solution of dopamine results in the deposition of a thin PDA film that can react with any compound containing amine or thiol groups. However, the molecular mechanism of PDA accumulation is not fully understood due to its heterogeneity and adverse physical properties.^16^

Exploiting the unique properties of PDA, adhesion, and biocompatibility, it is emerging as a promising candidate for interfacial coating in microfluidic devices. In order to create reliable in vitro tumor models using microfluidic platforms, it is essential to achieve strong and long-lasting adhesion of the artificial matrices. In our model, we use type I collagen hydrogels that are seeded with pancreatic cancer cell lines at different stages of the epithelial to mesenchymal transition (EMT). These cell lines will have different interactions with the surrounding ECM, resulting in different levels of deformation and contraction of the matrix. The aim of this work is to increase the adhesion and stability of these 3D hydrogels to improve the structural integrity of the matrix, addressing a critical issue prevalent in current methods: the propensity of hydrogels to contract and detach due to mechanical forces exerted by highly invasive pancreatic cancer cell lines. The results of this study, which demonstrate the improved stability and viability of 3D cultures facilitated by PDA coatings, have significant implications for the development of 3D culture models for cancer research. Establishing a platform that enables long-term and stable 3D culture is essential for studying the behavior, dynamics, and responses of pancreatic cancer cells in a controlled microenvironment. In addition, the knowledge gained from this work could potentially pave the way for advances in drug screening, personalized medicine, and the wider field of cancer therapeutics.

## 2 Materials and Methods

### 2.1 Experimental Set Up and Functionalization of Microfluidic Devices

**Figure 1:**
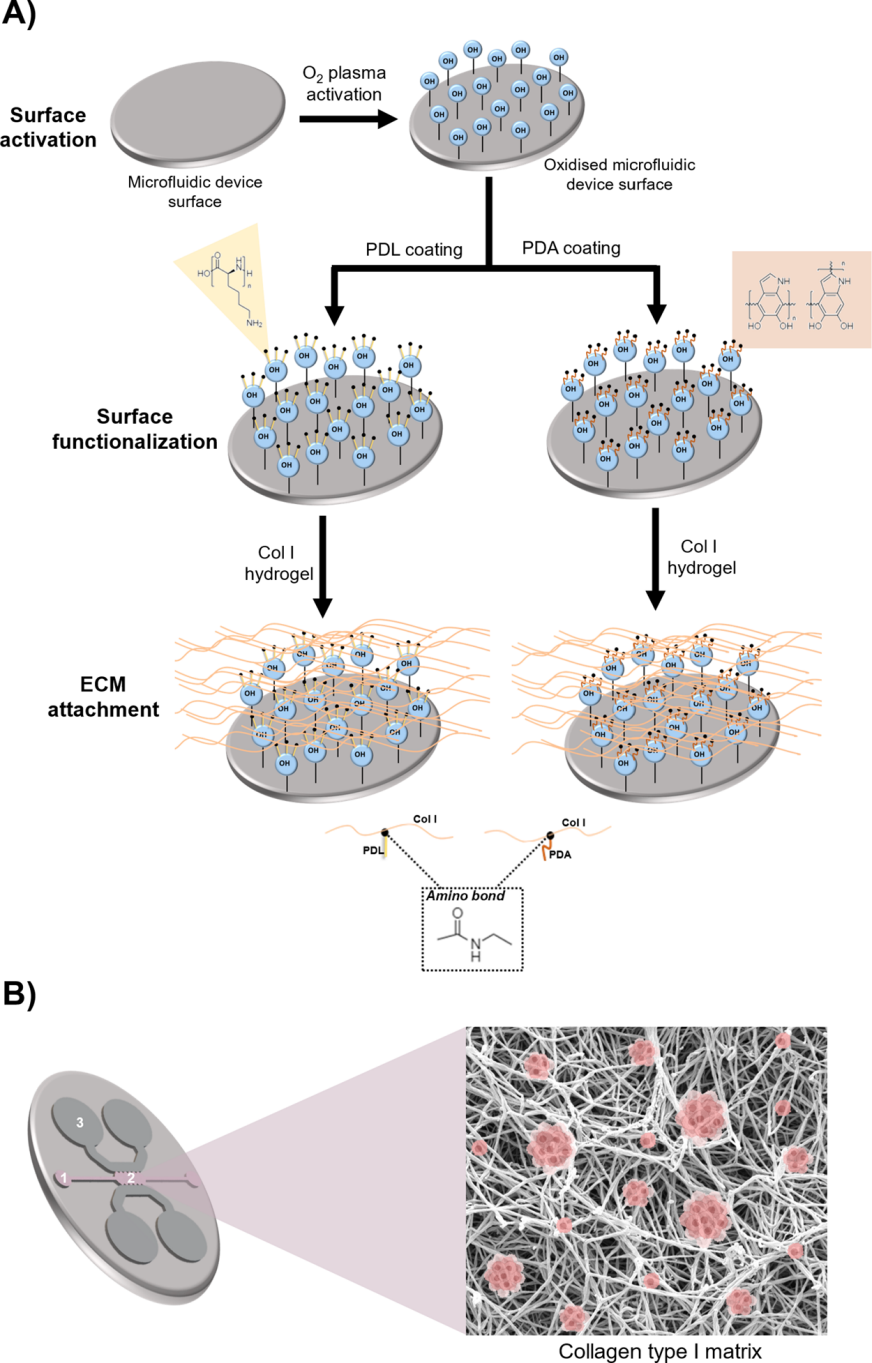
Experimental set up. (A) Activation and functionalization of PDMS microfluidic devices for long-term 3D culture. Polydimethylsiloxane (PDMS) microfluidic devices were functionalized first by O_2_ plasma surface activation, followed by coating with either poly-d-lysine (PDL) or polydopamine (PDA). (B) Schematic of the microfluidic device. Single pancreatic cancer cells embedded in a type I collagen hydrogel are introduced through the loading port (1) into the central chamber of the device (2). Through the reservoirs (3), the culture medium is introduced. The isolated cells self-organize three-dimensionally, interacting with the matrix and generating tumor spheroids from single cells.

Polydimethylsiloxane (PDMS) microfluidic devices are functionalized after patterning a PDMS substrate by replica molding from a master mold (Section 2.3). The PDMS surface is activated by the O_2_ plasma treatment to introduce functional groups through an oxidation reaction. O_2_ plasma removes organic hydrocarbon material by chemical reaction with highly reactive oxygen radicals and ablation by energetic oxygen ions. This leaves silanol (SiOH) groups on the surface, rendering the surface more hydrophilic and increasing surface wettability. Following plasma activation, the PDMS is immediately placed in contact with another oxidized PDMS or glass surface to form a bridging Si–O–Si bond at the interface, creating an irreversible seal. This water-tight covalent bond is ideal for microchannel formation and function. The PDMS or glass surface is then functionalized to facilitate the adhesion of extracellular matrix components, such as type I collagen. In this work, functionalization is performed using a coating of poly-d-lysine (PDL) or polydopamine (PDA). Type I collagen (Col I) hydrogel solution with the embedded PDAC cells is then introduced into the functionalized devices as described in Figure 1B and incubated for up to 7 or 11 days at 37 °C and 5% CO_2_. Collagen interacts with PDL or PDA molecules through an amino bond, which ensures the adhesion of the hydrogels to the PDMS surface of the devices. The schematic process of activation and functionalization of the PDMS microfluidic devices is shown in Figure 1A.

### 2.2 Cancer Cell Lines Culture

In this study, we obtained three pancreatic cancer cell lines, BxPC-3, Capan-2, and Panc-1, from the American Type Culture Collection (ATCC, USA). These cell lines were cultured in Dulbecco’s modified Eagle medium (DMEM, Gibco, Spain) with high glucose concentration (4.5 g/L d-glucose, l-glutamine, pyruvate) and supplemented with 10% fetal bovine serum (FBS, Life Technologies, Spain). However, the Capan-2 cell line required a higher concentration of serum (20% FBS) to maintain optimal growth. All of the cells were maintained under standard cell culture conditions, including a humidified atmosphere incubator set at 37 °C and 5% CO_2_.

### 2.3 Fabrication of Microfluidic Devices

The microfluidic devices used in this study were based on a design mentioned previously^17,18^ and consisted of a central chamber (2.5 × 1.3 mm) containing an array of trapezoidal posts to cage the collagen hydrogel solution with the embedded cells and two parallel side channels as medium reservoirs with a height of 300 μm.

Microdevices were fabricated according to Shin et al.^19^ using PDMS (Dow Corning) and a soft photolithography technique. The PDMS microdevices were autoclaved and bonded to a 35 mm glass-bottom Petri dish (Ibidi) by plasma treatment. They were then coated with poly-d-lysine hydrobromide (PDL, Sigma-Aldrich, Spain) at 1 mg/mL in cell culture water (Gibco, Spain) and different polydopamine (PDA, Sigma-Aldrich, Spain) solutions (0.5, 1, and 2 mg/mL) in 10 mM Tris-HCl pH 8.5 to better enhance type I collagen adhesion onto the channel surface. After washing and drying overnight in a dry oven at 80 °C (PDL)/60 °C (PDA) to restore the hydrophobicity of the bonded surface, the microfluidic devices were ready to use.

### 2.4 Loading of Hydrogel and Cells Into Microfluidic Devices

Hydrogels were made by mixing rat tail collagen type I (Corning, Spain) with high glucose DMEM medium, 10× DPBS, and 0.5 M NaOH solution (both from Sigma-Aldrich, Spain) at 2.5 and 4 mg/mL (pH 7.4), following Shin et al.^19^ Pancreatic cancer cells, as isolated cells, were mixed with the collagen solution, pipetted into the central chamber (approximately 750 cells/device) through the loading ports (Figure 1B), and incubated for 20 min for polymerization at 37 °C. The hydrogels were then hydrated through the reservoir ports (Figure 1B) with high-glucose DMEM. The culture is maintained for 7–10 days with manual medium renewal every 48 h through the reservoir ports.

### 2.5 Cytotoxicity

Cell viability was determined using the MTT assay. Briefly, cells were seeded at a density of 1 × 10^4^ cells/well in 96-well tissue culture plates (Avantor VWR, Spain). These plates have a vacuum-gas plasma treatment for consistent cell attachment and growth. After plasma activation, they were treated with the coating solutions studied in this work, following the same protocol as explained in Section 2.3*.* They were incubated for 6 days to evaluate the cytocompatibility of the used coating substances. At days 1, 3, and 6, MTT solution (5 mg/mL, Sigma-Aldrich, Spain) was added to each well, and the plates were incubated for an additional 4 h at 37 °C. The supernatants were then removed, and the formazan crystals were solubilized in DMSO. The absorbance was measured at 570 nm using a microplate reader (Synergy LX, BioTek with Gen5 3.10 software).

### 2.6 Imaging and Spheroid Growth Quantification

Growth and morphology of the 3D PDAC spheroids were monitored for 10 days by acquiring phase-contrast images every 48 h with an inverted optical microscope (DM IL LED, Leica, Wetzlar, Germany). The estimated size and shape of tumoral cells and spheroids were analyzed using a semiautomatic hand-coded script for segmentation with MATLAB (Mathworks, Natick, CA, US), based on active contours. Figure S1 in the Supporting Information shows an example of this self-developed application while segmenting a particular image. Using the cell counting software integrated into the Countless 3 automated cell counter (Invitrogen), mean diameter measurements of the cell lines used in this study at the initial time of seeding were obtained. These data were used to calculate the individual cell area of each cell line (Table S1). Any segmented object with an area greater than these values was considered to be a cellular aggregate capable of forming a spheroid from a single cell during the culture period inside the microfluidic device.

### 2.7 Immunofluorescence Staining

The PDAC spheroids were stained inside the microfluidic device with DAPI and phalloidin, and images of the stained spheroids embedded in the collagen hydrogel inside the device were taken using a Lattice Lightsheet 7 microscope equipped with a 40× objective (Zeiss, Germany).

The cells inside the microfluidic device were fixed in 4% paraformaldehyde (Sigma-Aldrich, Spain) for 20 min at room temperature, washed three times with PBS, and permeabilized for 15 min with PBS 0.1% Triton-X100 (Calbiochem, Spain). The cells were washed three more times with PBS and incubated with PBS 5% bovine serum albumin fraction V (BSA, Merck, Spain) solution overnight at 4 °C on a rocker. Afterward, they were incubated for 4 h at room temperature in the dark with Phallodin-TRITC 1:100 to stain actin cytoskeleton (0.125 mg/mL, ChemCruz, USA) and DRAQ5 20 μM for staining of cell nuclei (5 mM, Thermo Scientific, Spain). Finally, the samples were washed 3 times with PBS and stored at 4 °C until imaged.

### 2.8 Data and Statistical analysis

All tests presented in this study were carried out in duplicate. The data and statistical analysis from the MTT assay and the segmentation analysis (spheroid area and eccentricity) were carried out using the statistical software GraphPad Prism v8.0.1 in combination with Microsoft Excel software. First, the normality of the data was assessed with the Shapiro-Wilk or Kolmogorov tests, depending on the number of data. Also, Levene's test was performed to analyze the homogeneity of the variance. After, normality and homogeneity of the variance test, analysis of variance (ANOVA) was performed to determine statistical significance among the studied continuous variables in the different conditions. Depending on the information obtained from the normality and homogeneity of variance analyses, different parameters for the ANOVA test were set: Welch ANOVA with Games-Howell as posthoc, ordinary ANOVA followed by post hoc Tukey–Kramer tests, Kruskall-Wallis tests with posthoc Dunn's test. In some cases, a *t*-test was used, followed by a Mann–Whitney *U* test. A *p-*value (α) below 0.05 was considered a significant result. Results presented in violin plots show median, quartiles (Q1 and Q3), and maximum and minimum values.

## 3 Results and Discussion

### 3.1 Assessment of Collagen Hydrogel Adhesion

**Figure 2:**
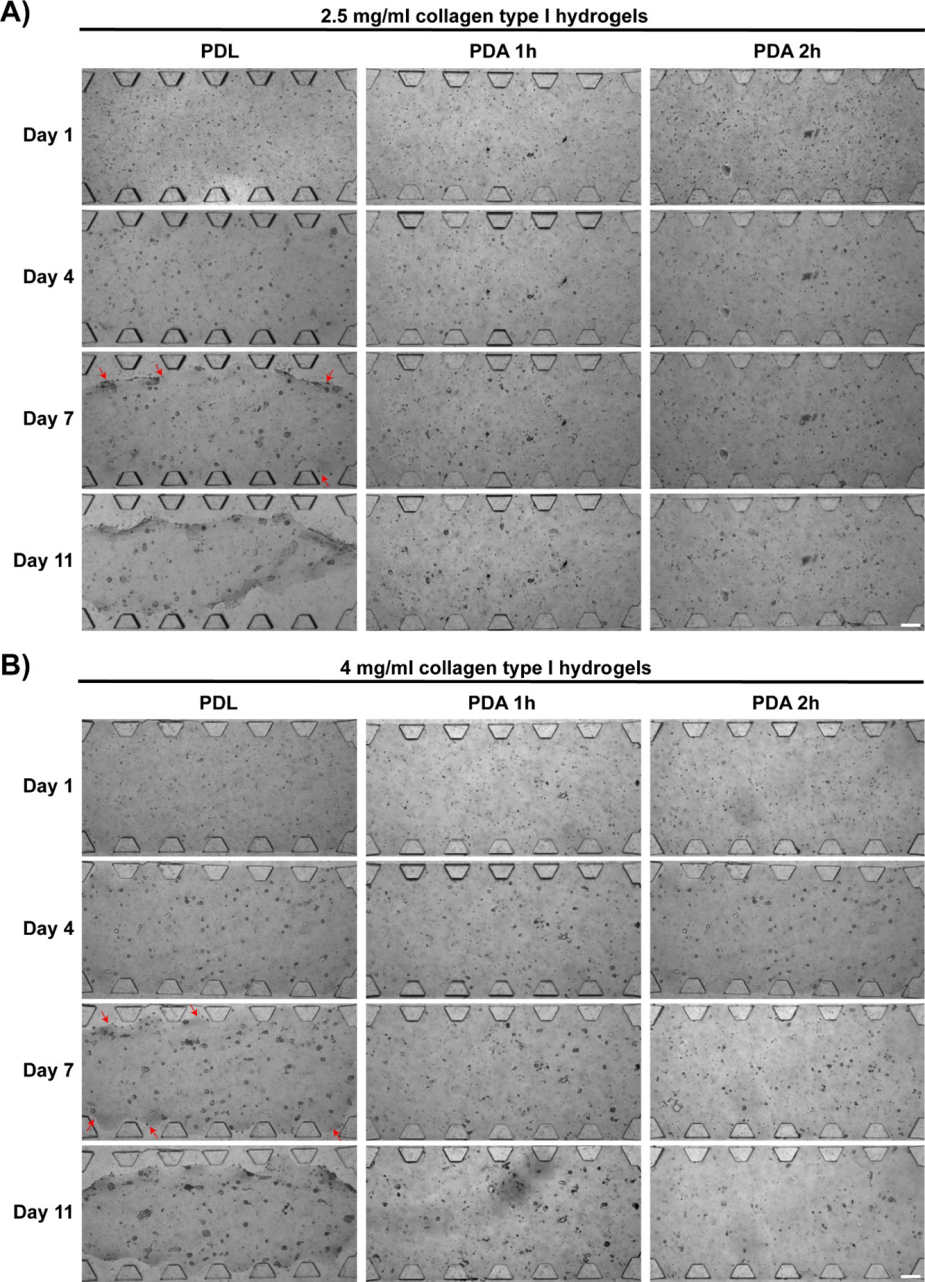
Collagen hydrogel adhesion to different interfacial coatings: PDL (poly-d-lysin), PDA 1 h (polydopamine for 1 h) and PDA 2 h (polydopamine for 2 h). Images show brightfield microscopy of the central chamber of the microfluidic devices used in this study. The PDAC cell line shown is BxPC-3. (A) 2.5 mg/mL collagen type I hydrogel. (B) 4 mg/mL collagen type I hydrogel. It can be easily observed that hydrogels in devices coated with PDL detached by day 7 of culture (the red arrows point to the hydrogel detachment sites), while those in the PDA coated devices remained in place for the duration of the experiment. Scale bar = 200 μm.

**Figure 3:**
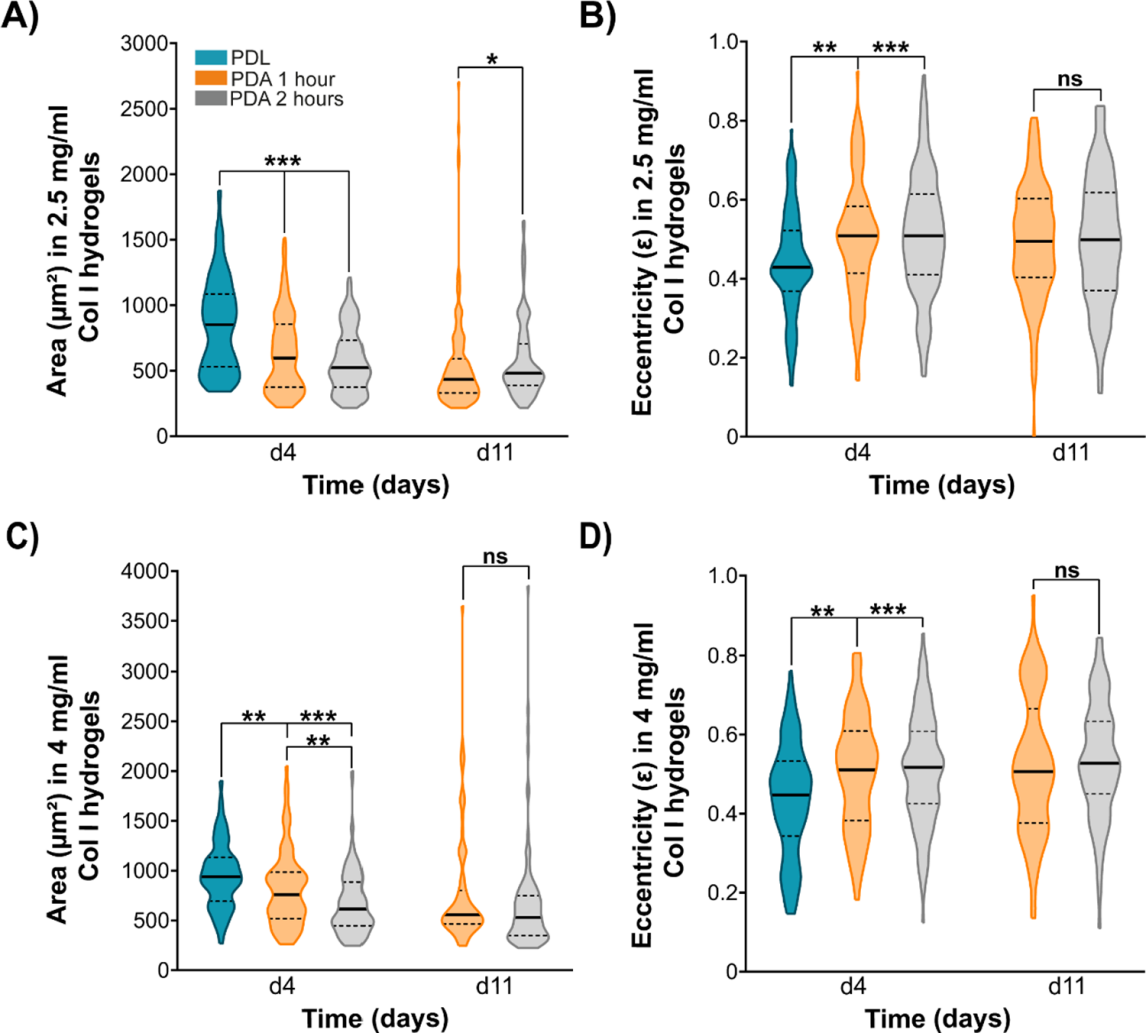
Quantification of BxPC-3 spheroid size and morphology in type I collagen hydrogels in the different coatings studied for the microfluidic device. Comparison of the spheroid size and morphology of the BxPC-3 cell line on day 4 and day 11 in 2.5 mg/mL (A, B) and 4 mg/mL (C, D) collagen hydrogels in microfluidic devices with different coating treatments. The morphological characterization of the spheroids was done using the quantification of the eccentricity parameter. Spheroid growth in the PDL-coated devices in both hydrogels could only be analyzed up to day 4 due to detachment of the hydrogels from the central chamber. *p* value reported in APA style: 0.12 (ns), 0.033 (*), 0.002 (**), < 0.01, (***).

**Figure 4:**
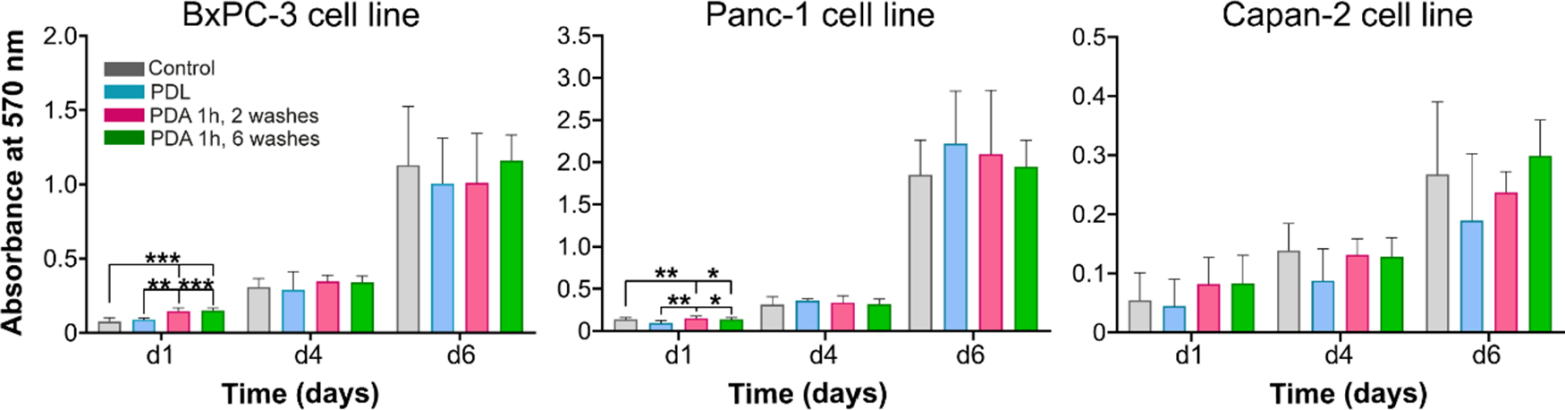
Cytotoxicity of PDA coatings for BxPC-3, Panc-1, and Capan-2 cell lines. Results show mean and standard deviation of the mean (SD) of two independent experiments with 3 replicates. P value reported in APA style: 0.12 (ns), 0.033 (*), 0.002 (**), < 0.01, (***).

The use of PDA to enhance the adhesion of ECM hydrogels has been investigated in previous studies involving other types of 3D cultures, demonstrating significant benefits in increasing the adherence of these matrices in vitro even during prolonged culture periods.^20^ To refine the application of this molecule in our microfluidic-based 3D culture model, we initially assessed the effects of PDA coating (2 mg/mL) incubation time (1 or 2 h) on the adhesion of 2.5 and 4 mg/mL rat tail collagen type I hydrogels in our microfluidic devices, and we performed a 3D culture with the BxPC-3 cell line until day 11. Cell-free collagen hydrogels can adhere to the surface of devices for extended periods of time when treated with the PDL coating (not shown). However, this cell line generates spheroids whose contractile forces are able to detach the hydrogels off the microfluidic device surface when treated with PDL. The polydopamine coating improved the long-term adhesion of collagen hydrogels (Figure 2). It was observed that the hydrogels remained well confined in the device until day 11, while the devices treated with PDL presented detached hydrogels due to the forces generated by the spheroids of this cell line on the ECM. This effect was observed at both the collagen concentrations tested, i.e., 2.5 mg/mL (Figure 2A) and 4 mg/mL (Figure 2B). Impeded detachment of the hydrogels in the PDA coatings could be related to increased adhesion to the surface of the devices. No incubation time-dependent differences in adhesion were observed. However, differences in spheroid formation and development were seen when comparing PDL and PDA coatings. Given these differences, we quantified the growth and characterized the morphology over time of BxPC-3 spheroids in the different coatings used (Figure 3).

Qualitative and quantitative differences are observed in the formation and growth of 3D spheroids of the BxPC-3 cell line, depending on the coating used on the microdevices. This cell line can generate larger spheroids over time in PDL-coated versus PDA-coated microdevices. Specifically, the BxPC-3 cells show the ability to generate slightly larger spheroids with regular morphology over time on PDL-coated microdevices compared to PDA-coated microdevices in both matrices (Figure 3). These differences may be attributed to the varying adhesion properties between the matrix and the microdevice surface. With PDA coatings, we observed stronger adhesion of the hydrogels to the device surface, which may be causing the tumor cells to sense a different mechanical environment compared to the weaker adhesion observed with PDL coatings. This appears to influence the development of the spheroids, resulting in a smaller size and more irregular morphology (Figure 3) when encountering hydrogels with stronger adhesion to the surface. Notably, these differences manifest primarily in the early stages of spheroid development due to the detachment of the matrix observed with PDL coatings (Figure 3). Upon extension of the culture duration, significant variations in spheroid size become evident on PDA-coated matrices with a collagen concentration of 2.5 mg/mL, depending on the incubation time (Figure 3A). In contrast, the spheroid morphologies remain consistent (Figure 3B). Importantly, these size differences are minimized with increasing collagen concentration in the matrix (Figure 3C).

The observation of differences in the size and morphology of BxPC-3 spheroids when cultured in microfluidic devices coated with PDL or PDA coatings at early time points prompted us to re-evaluate the experimental protocol. Since the incubation time of PDA did not affect our PDAC cell model, the time incubation of 1 h for PDA has been chosen as sufficient time to ensure better adhesion of the hydrogels to the microdevices, allowing the correct development of the spheroids at longer culture times compared to the PDL coating. Although the incubation time of PDA was not found to be a significant factor for the adhesion of the collagen hydrogels and the BxPC-3 spheroid formation, we assessed the impact of device coating washes on the two factors mentioned above. Our microfluidic devices are typically washed six times with H_2_O to mitigate PDL cytotoxicity and remove any excess PDL present in the device. PDA coatings have been shown to exhibit exceptional biocompatibility in different cell types due to the presence of its precursor in the human body.^21,22^ Various studies have investigated the adhesion and behavior of diverse cell types on PDA-coated surfaces, revealing that the compatibility of PDA coatings with cells is dependent on the specific cell type.^23^ However, the exact mechanism for these compatibility variations with different cell types remains undetermined.^15,20^ We analyzed its cytocompatibility with the PDAC cell lines used for our microfluidic model. Results show that at long culture times, there were no statistically significant differences found among the four experimental groups, namely, control (no coating), 1 mg/mL of PDL, and 2 mg/mL of PDA incubated for 1h and washed 2 times (PDA 2 washes) or 6 times (PDA 6 washes), for any of the cell lines employed. In all tested coatings, we observed similar growth of the three cell lines compared to their uncoated control (Figure 4). Therefore, we can conclude that the PDA coating used in this study has no cytotoxic effects on the cancer cell lines tested.

**Figure 5:**
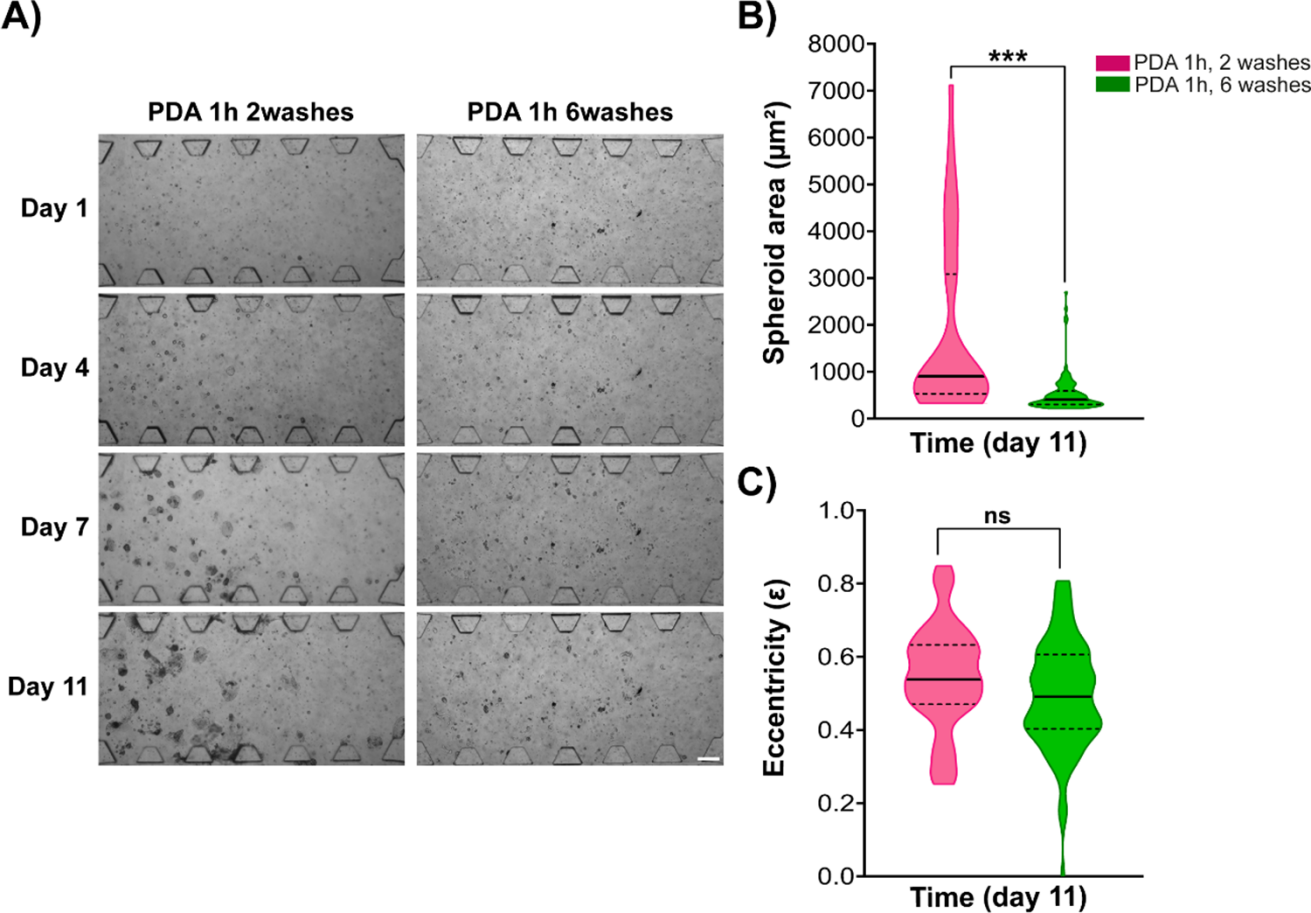
BxPC-3 spheroids in 2.5 mg/mL type I collagen hydrogels in microfluidic devices with PDA coatings incubated 1 h and washed 2 or 6 times. (A) Images show brightfield microscopy of the BxPC-3 spheroids in the central chamber of the microfluidic devices used in this study. Scale bar is 200 μm. (B) End point quantification of BxPC-3 spheroid size in type I collagen hydrogels in the different washes conditions of the PDA coating studied for the microfluidic device. (C) Characterization of the spheroid morphology at day 11 of the BxPC-3 cell line on PDA-coated devices, washed 2 or 6 times. p value reported in APA style: 0.12 (ns), 0.033 (*), 0.002 (**), < 0.01, (***).

Since PDA is a biocompatible material and does not display cytotoxic effects in 2D cultures regardless of the number of washes (Figure 4), we evaluated if we could decrease the number of washes in our devices while still achieving positive outcomes in BxPC-3 spheroid development in 2.5 mg/mL collagen type I hydrogels. Differences were observed in the size of the spheroids generated in the 2.5 mg/mL type I collagen hydrogels using microfluidic devices coated with PDA (2 mg/mL, 1 h incubation) after being washed two or six times over time (Figure 5B). For medium-term culture, the formation and development of spheroids of this cell line were significantly better when only two washes were performed (Figure 5A). Therefore, based on these results and those obtained from PDA incubation times (Figure 3), we concluded that the use of a PDA solution of 2 mg/mL incubated for 1 h at room temperature, followed by a couple of subsequent washes of the devices with H_2_O was suitable coating conditions for our type of 3D microfluidic-based culture.

However, we still observed some differences in the morphology and size of the spheroids formed in PDA coatings versus in PDL coatings. Several studies have confirmed the biocompatibility of polydopamine with various cell lines.^23^ The present study has further shown that this substance, when used as a coating, does not exhibit cytotoxicity toward the cell line being studied. Therefore, the differences observed in spheroid generation using two types of coatings could be attributed to a mechanical rather than a chemical cause. The mechanical forces exerted by cells and the tension of the ECM are crucial in establishing and maintaining tissue homeostasis.^24^ Cell function depends on the rigidity of the ECM, which cells probe by applying and transmitting forces to it and then transducing them into biochemical signals. In the context of tumor development and progression, the forces exerted by tumor cells on the ECM play a significant role. These forces can modify the behavior of both tumor cells and the ECM itself, dynamically adapting by modifying its behavior and remodeling its microenvironment.^25^ This emphasizes the importance of the mechanical environment in cancer spheroid formation.^26,27^ Our preliminary results show that by enhancing the adhesion of collagen to the PDMS surface, the same force applied by cells on the ECM as in PDL coatings can no longer deform the hydrogel to the same extent as before. This suggests that the forces exerted by tumor cells on the ECM are influenced by the strength of adhesion of the hydrogel to the coated surface of the microfluidic devices. This difference in adhesion strength may lead to variations in the deformation of the matrix and subsequently affect tumor growth. These findings align with the understanding that the mechanical microenvironment plays a prominent role in tumor development. Studies have shown that alterations in the mechanical properties of the ECM can influence tumor cell contractility, invasion, and metastasis.^28^ Additionally, the remodeling of the ECM by tumor cells can further modulate the mechanical microenvironment and impact tumor growth.^22^ Consequently, this alteration in the hydrogel adhesion seems to modify the cells’ mechanical environment and affects the formation and development of tumor spheroids. These findings highlight the importance of considering the mechanical microenvironment, including the ECM properties and cellular forces in understanding tumor growth and phenotypic responses.^29,30^

### 3.2 Fine-tuning PDA Coating for Optimal PDAC Spheroid Growth

To minimize the mechanical stress experienced by the spheroids on the PDA-coated microfluidic devices while maintaining hydrogel-favorable adhesion, we reduced the PDA concentration in the coating solution while keeping the other optimal conditions unchanged.

**Figure 6:**
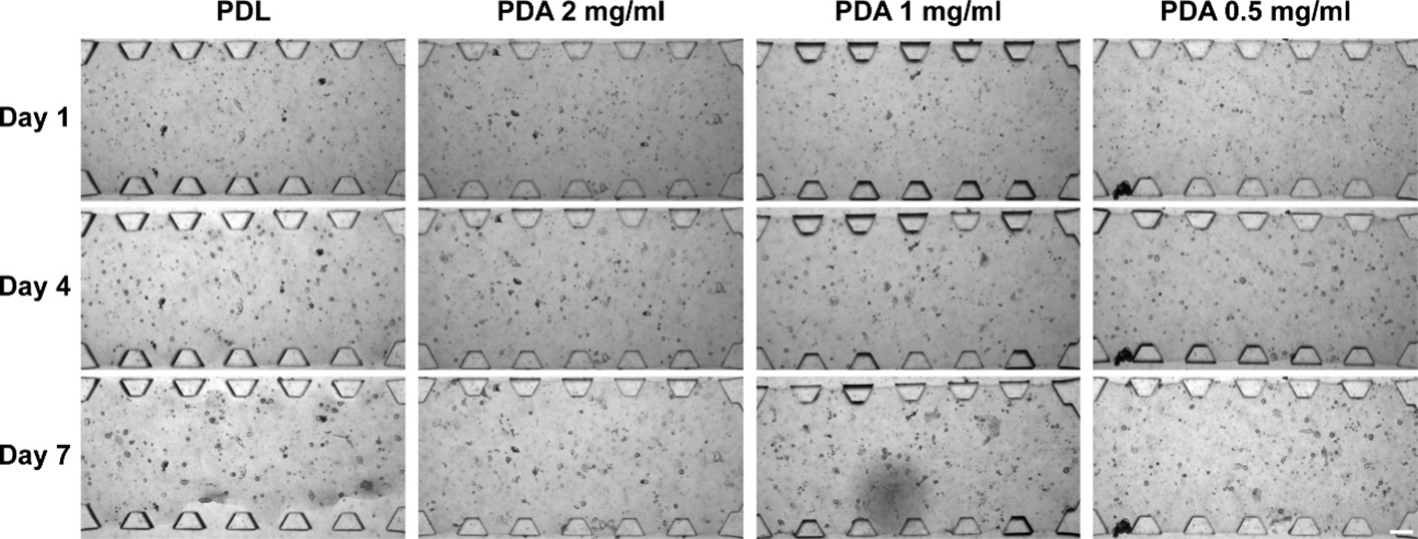
Collagen hydrogel (2.5 mg/mL) adhesion and BxPC-3 spheroid development over time in different microfluidic device coatings: PDL 1 mg/mL and PDA at 0.5, 1, and 2 mg/mL. Images show bright-field microscopy of the BxPC-3 spheroids in the central chamber of the microfluidic devices embedded in type I collagen hydrogels of 2.5 mg/mL. Scale bar is 200 μm.

**Figure 7:**
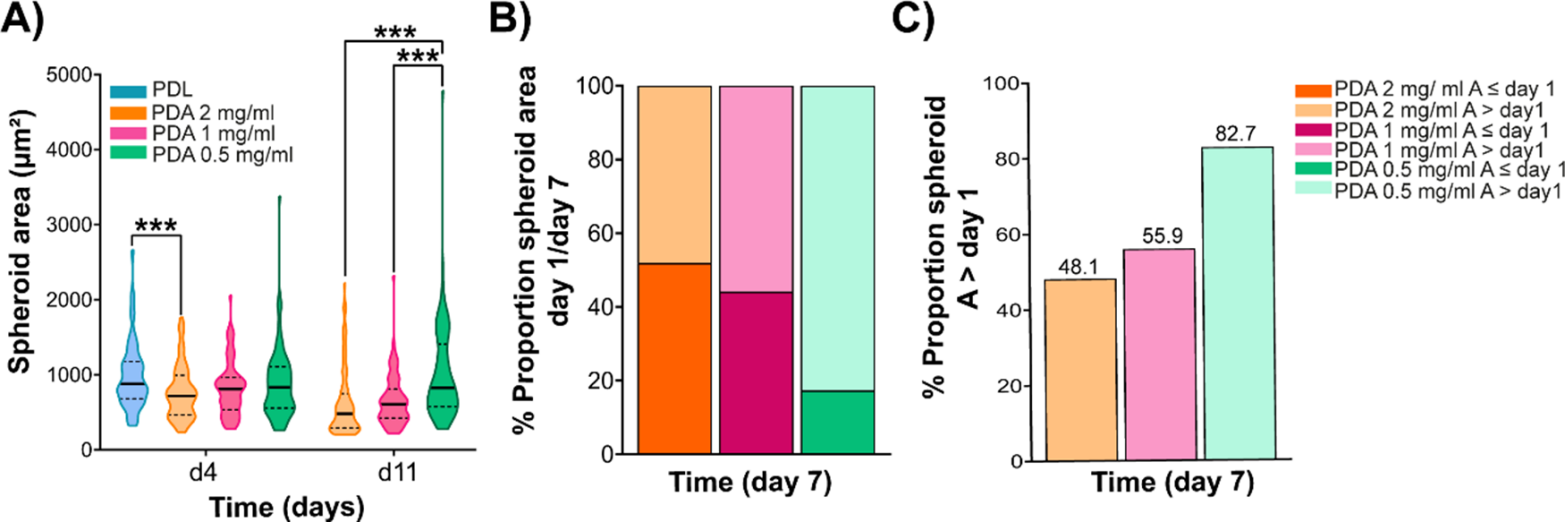
Quantification of the size of BxPC-3 spheroids in type I collagen hydrogels using different concentrations of PDA in the coating solutions of microfluidic devices. Spheroid’s growth was monitored over time in 2.5 mg/mL type I collagen hydrogels. (A) Size growth of BxPC-3 cell line spheroids at day 4 and 11 in the different coating conditions. (B) Percentage of spheroids classified based on their area at day 7 relative to the average area on day 1 of cell culture. (C) Final time comparison of the percentage of spheroid population with an area above day 1 area average. p value reported in APA style: 0.12 (ns), 0.033 (*), 0.002 (**), < 0.01, (***).

Reducing the concentration of polydopamine in the coatings had no impact on the adhesion of the collagen hydrogels, as all maintained their integrity until the endpoint (Figure 6). However, differences in spheroid generation were observed depending on the concentration of PDA used to coat the devices. We observed that a higher concentration of PDA (2 mg/mL) leads to the formation of smaller spheroids at day 7 compared to those generated using lower concentrations of PDA (Figure 7A). At shorter culture times, devices coated with PDL and 0.5 mg/mL PDA showed more similar growth compared to those coated with 1 and 2 mg/mL PDA solutions (Figure 7A). On the other hand, we observed that with increasing PDA concentration, the number of cells able to grow into spheroids larger than the area of the cell observed at initial culture times is lower (Figure 7B). Only when using the PDA concentration of 0.5 mg/mL, a higher percentage of cell populations were observed to develop spheroids with an area larger than the average area of cell clusters obtained on day 1 of culture (Figure 7C). This suggests that the long-term development of BxPC-3 cell line spheroids is compromised with the increasing PDA concentration. This phenomenon may be related to the previously proposed hypothesis that the adhesion of the hydrogel to device surfaces is related to mechanical forces exerted by cells within the collagen matrix. In high-concentration PDA-coated conditions, where the collagen matrix seems to remain relatively more adherent to the surface, cells may experience increased resistance when trying to deform the matrix. This strong resistance seems to affect the proliferative capacity of the cells, preventing the proper development of spheroids. In contrast, in lower concentration PDA-coated and PDL-coated environments, where matrices may be less adherent to the surface, cells may find it easier to physically shape the matrix to accommodate their growth to self-organize three-dimensionally into tumor spheroids. Cellular stiffness sensing depends on intracellular tension, which results from the balance of forces generated by the contractile cytoskeleton and the elastic resistance (stiffness) of the ECM.^31^ Our results show how this tension appears to be a critical determinant in spheroid morphology and growth dynamics in cell lines capable of exerting highly contractile forces on the surrounding matrix.

Based on our analysis, we determined that the optimal conditions for coating our devices involve the use of a PDA solution with a concentration of 0.5 mg/mL. The coating solution should be incubated for 1 h at room temperature, followed by moderate rinsing of the devices (twice) with H_2_O. These optimized conditions ensure an efficient and reliable adhesion of the type I collagen hydrogels and allow a balance between the adhesion of the hydrogels and the deformation forces that the spheroids are able to generate on them, favoring the correct development and 3D growth of the spheroids on our devices.

**Figure 8:**
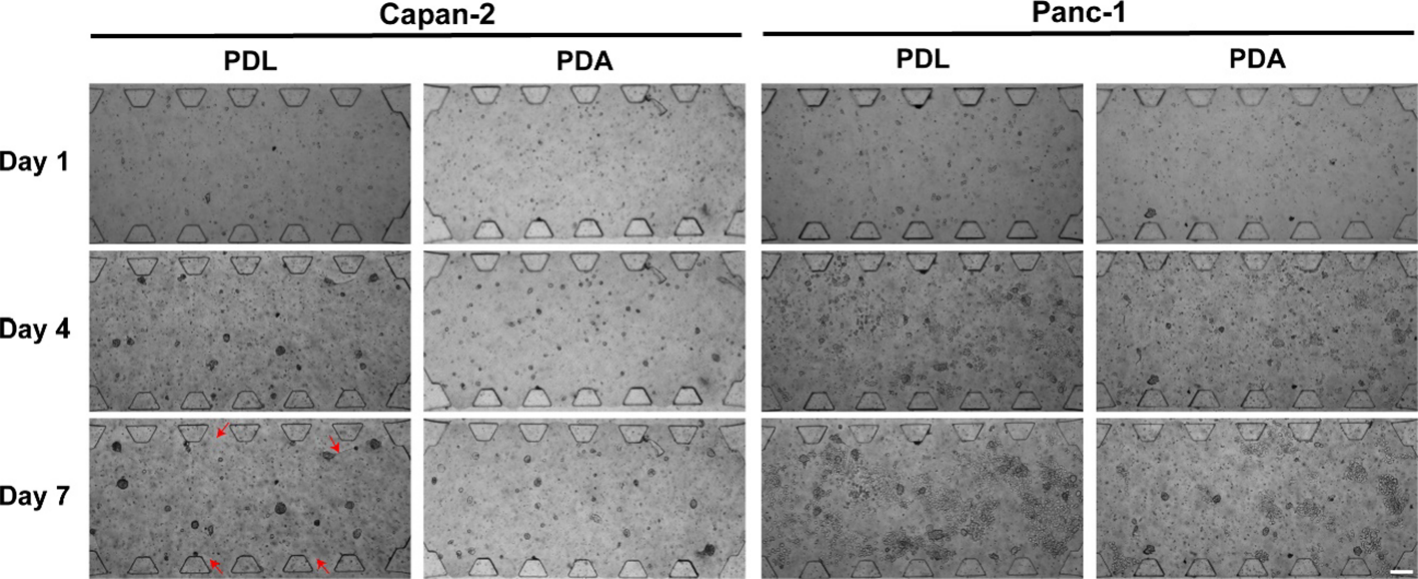
Time tracking of the adhesion of collagen type I hydrogel and spheroid development in Capan-2 and Panc-1 cell lines into the microfluidic devices coated with 0.5 mg/mL of PDA solution. Brightfield images of the central chamber of the microfluidic devices with the cell line spheroids embedded in the collagen hydrogel. The red arrows show the hydrogel detachment from the surface of the device. Scale bar 200 μm.

To further validate the impact of the coating on PDAC cell lines, we evaluated the optimal conditions obtained with two additional pancreatic cell lines with different genetic complexity and different grades of neoplastic differentiation (Capan-2 and Panc-1). Capan-2 cells share similar features to the BxPC-3 cell line when cultured in PDL-coated devices, with spheroids exerting substantial forces that hinder the adhesion of low collagen type I hydrogels to our microfluidic platforms (Figure 8). In contrast, the Panc-1 cell line forms spheroids that demonstrate minimal hydrogel detachment caused by forces on the ECM, making it an appropriate morphological and growth control for our experiments.

**Figure 9:**
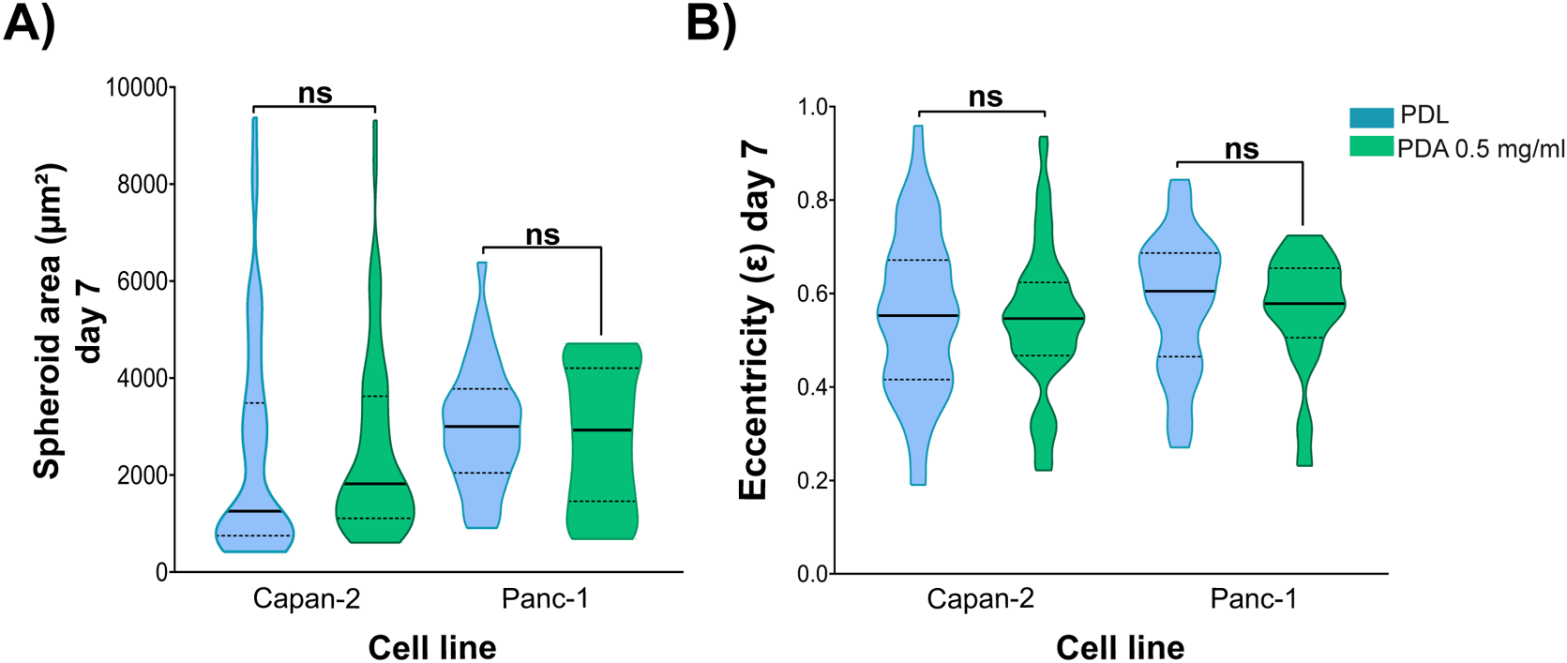
Characterization of the size and morphology of spheroids of Capan-2 and Panc-1 cell lines in microfluidic devices using an optimized PDA coating. (A) Quantification of the size of spheroids generated by Capan-2 and Panc-1 cell lines in 2.5 mg/mL type I collagen hydrogels in PDL- or PDA-coated devices. (B) Morphology of the spheroids generated by both cell lines in the different microfluidic devices according to the type of coating. All data shown represent the end point of the culture time (Day 7).

Our findings corroborate our previous observations with the BxPC-3 cell line. The use of a 0.5 mg/mL PDA solution to coat our microfluidic devices robustly enhances hydrogel adhesion. This, in turn, facilitates an extended spheroid culture, even under conditions involving considerable forces exerted on the ECM (Figure 8). Notably, both Capan-2 and Panc-1 cell lines generated spheroids with no significant differences in size and morphology on both coatings, PDL and PDA 0.5 mg/mL. (Figure 9A, B). This result demonstrates previous results obtained with the BxPC-3 line in which a PDA concentration of 0.5 mg/mL not only improves adhesion but also decreases mechanical stress on the spheroids, promoting their optimal development within our devices over prolonged time periods.

### 3.3 Immunostaining of Generated Spheroids Grown in Optimal Conditions

**Figure 10:**
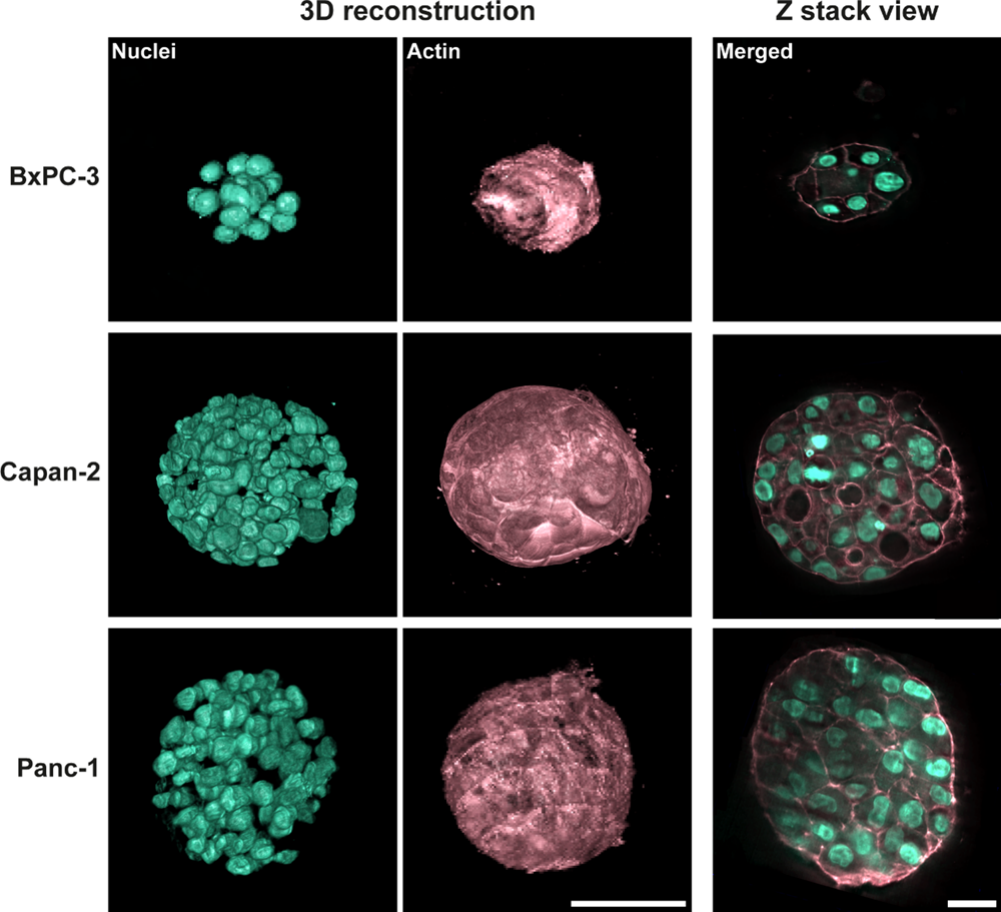
3D reconstruction of the structure of spheroids generated by Panc-1, Capan-2, and BxPC--3 cell lines in 2.5 mg/mL collagen type I matrices in PDA-coated (0.5 mg/mL) microfluidic devices. 3D reconstruction and Z-stack view (2D) of fluorescent lattice lightsheet (scale bar of 50 and 20 μm, respectively, 40× magnification) images of PDAC spheroids after 7 days of growth.

The last part of our study involved close observation of the structure of spheroids generated by the three cell lines used in this study, grown in the optimal conditions already described. For this purpose, we performed an immunofluorescence staining of the spheroids embedded in the collagen type I hydrogel with DAPI and phalloidin. The samples were observed inside our microfluidic device under a fluorescent lattice lightsheet microscope, which allows 3D reconstruction of the obtained images for individual spheroids (Figure 10). The DAPI staining allowed us to visualize cell nuclei, offering insight into the compactness of spheroids. Additionally, phalloidin staining provided insights into the actin cytoskeleton, revealing intriguing patterns of the cell shape and arrangement within the spheroids. Our results showed that PDAC cells are able to self-organize three-dimensionally embedded in collagen-type I hydrogels in microfluidic devices, forming compact and well-organized spheroids with distinct cell–cell interactions.

The 3D reconstruction of the spheroids provided a complete picture of their internal architecture, thanks to which we observed differences in the spheroid structure between the three cell lines. These observations revealed distinctive features that can be attributed to the specific characteristics of each cell line and its mechanical adaptation. The BxPC-3 cell line forms spheroids with a smaller number of cells, while the Capan-2 and Panc-1 lines generate more proliferative spheroids. In all cases, the common feature is the formation of spheroids, where the cells seem to have a less compact organization. This fact would corroborate the morphology data observed in the spheroids generated under the optimized conditions of this study (Figures 7 and 9), in which the three lines generated spheroid populations with a median eccentricity close to 0.5, which would denote some irregularity in their growth. This morphological feature can be attributed to the use of low-collagen matrices, whose mechanical and structural characteristics such as lower stiffness and larger pore size favor cell migration and invasion.^32^ Cells grown in these matrices tend to encounter less physical resistance as they move through the larger pores, allowing them to explore a greater spatial extent and potentially facilitating the growth of spheroids whose cells are less tightly packed.^4,26^ The low stiffness matrices are utilized to create models that enable the study of the mechanisms governing the mechanical invasiveness of tumor cells in 3D environments with varying mechanical properties. The differences observed in the number of cells found in the spheroids generated by the different cell lines analyzed suggest a relationship between the proliferative capacity of the cells and their mechanical adaptation to the surrounding microenvironment. The enhanced adhesion of collagen hydrogels achieved by optimal PDA coating conditions could be influencing the mechanical signals that cells encounter during spheroid development.^33^ Further studies are needed to confirm these results. Overall, the combination of the enhanced adhesion of the PDA coating and the observed spheroid structures suggests a strong interaction between mechanical signals and cell behavior.^34^ The optimal conditions of the PDA coating offer the opportunity to create a more representative and dynamic 3D microenvironment for spheroids, allowing a better understanding of the mechanical environment influence on tumor biology and the optimization of tumor development and invasion in vitro models for cancer research.

## 4 Conclusions

In this study, we extensively characterized the utilization of two chemical compounds, poly-d-lysine (PDL) and polydopamine (PDA) as coatings on activated surfaces to enhance the adhesion of collagen-based 3D extracellular matrices to PDMS microfluidic devices. The results have shown the remarkable efficacy of PDA coatings in significantly improving the adhesion of type I collagen hydrogels onto PDMS microfluidic devices, enabling the long-term cultivation of pancreatic cancer cell spheroids capable of exerting substantial contractile forces on the matrix during their development. Despite the biocompatibility of the PDA compounds, we observed that increasing the PDA solution concentration and the number of device washings after coating had detrimental effects on spheroid development in the analyzed PDAC cell lines. To address this issue, we successfully employed a 0.5 mg/mL PDA solution and reduced the number of washes after coating, effectively preventing hydrogel detachment from the device surface due to the forces exerted by the generated spheroids. This approach achieved a delicate balance between gel adhesion and spheroid contractile forces, which is crucial for optimal cell proliferation and long-term spheroid development.

Collectively, our findings offer a simple yet highly effective method for generating in vitro 3D culture models using microfluidic devices, particularly when working with cell lines capable of generating spheroids that exert significant forces on the surrounding ECM, such as PDAC cell lines. In response to the urgent need for the development of novel models for highly aggressive and metastatic solid tumors, this study has focused on optimizing PDA coatings for PDMS microfluidic devices. By enhancing the adhesion of extracellular matrices, particularly collagen hydrogels, these optimized PDA-coated devices allow for the prolonged culture of cancer spheroids, preventing matrix detachment while allowing prolonged cell-ECM interactions, providing a more physiologically relevant model for future studies. In summary, the optimization of PDA coatings on polydimethylsiloxane (PDMS) microfluidic devices, as presented in this study, significantly contributes to the advancement of novel in vitro tumor models. These 3D models not only represent a significant advancement in in vitro tumor research but also bridge the gap between traditional 2D cultures and the complex 3D nature of tumors in vivo*.* They faithfully replicate the inherent 3D attributes of tumor tissues, thereby enabling a comprehensive investigation into the intricate mechanical and biochemical interactions between tumor cells and the constituents of the TME throughout the course of tumor development. Microfluidic 3D tumor models offer a closer representation of in vivo tumor development, facilitating improved diagnostic and therapeutic strategies for these challenging malignancies.

## Data Availability

Data will be made available on request.

## Supporting Information Available

Additional details on materials and methods; table that summarizes the mean diameters and areas of the different PDAC cell lines as single cells; and figure that shows the interface of the Matlab segmentation app used for the analysis of spheroid growth and morphology (PDF)
