# Supplementary material for: Polydopamine Interfacial Coating for Stable Tumor-on-a-Chip Models: Application for Pancreatic Ductal Adenocarcinoma
Source: Biomacromolecules. 2024 Jul 31;25(8):5169–80. doi: 10.1021/acs.biomac.4c00551 (PMC11323005; doi:10.1021/acs.biomac.4c00551)
Supplement: Supplementary file 1 — bm4c00551_si_001.pdf [file bm4c00551_si_001.pdf]

# Supporting information

## Polydopamine interfacial coating for stable tumour-on-a-chip models: Application for pancreatic ductal adenocarcinoma

Soraya Hernández-Hatibi[1,2], Pedro Enrique Guerrero[1,2], José Manuel García-Aznar[1,3], Elena García-Gareta[1,3,4*]

1. Multiscale in Mechanical & Biological Engineering Research Group, Aragon Institute of Engineering Research (I3A), School of Engineering and Architecture, University of Zaragoza, Zaragoza, 50018, Aragon, Spain.
2. Department of Biochemistry and Molecular and Cellular Biology, Faculty of Sciences, University of Zaragoza, Zaragoza, 50009, Aragon, Spain.
3. Aragon Institute for Health Research (IIS Aragon), Miguel Servet University Hospital, Zaragoza, 50009, Aragon, Spain.
4. Division of Biomaterials & Tissue Engineering, UCL Eastman Dental Institute, University College London, London, WC1E 6BT, United Kingdom.

TABLE S1- Table with approximate values of the mean diameters and areas of the different PDAC cell lines as single cells. The mean value of the mean diameters of the live cells in 10 different counts was calculated with the automated cell counter software Countless 3.

| Cell line | Mean diameter (µm) | Mean Area (µm²) |
|---|---|---|
| **BxPC-3** | 15.5 | 201 |
| **Capan-2** | 16.1 | 201 |
| **PANC-1** | 18 | 254 |

Figure S1- Example of the Matlab segmentation app interface used for semi-automatic analysis of spheroid growth and morphology.

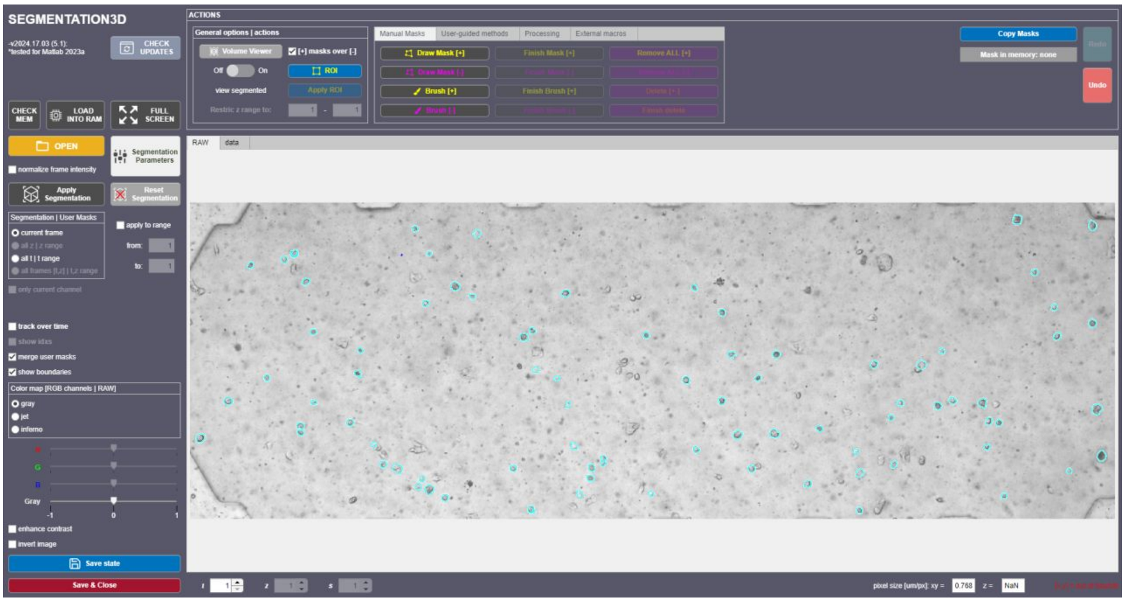

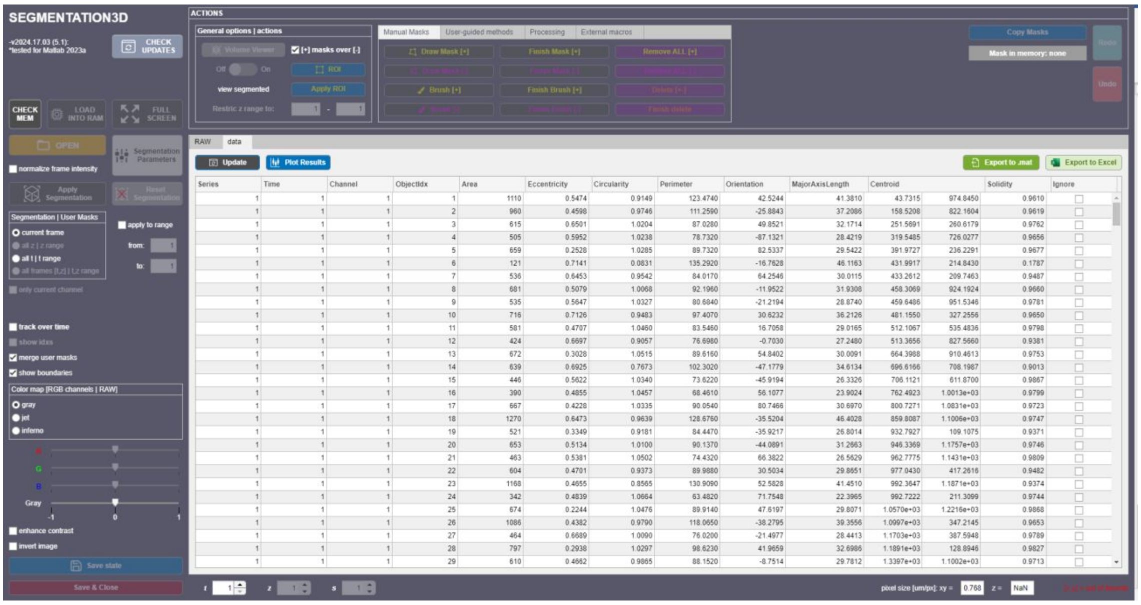


| Series | Time | Channel | ObjectIdx | Area | Eccentricity | Circularity | Perimeter | Orientation | MajorAxisLength | Centroid | | Solidity | Ignore |
|---|---|---|---|---|---|---|---|---|---|---|---|---|---|
| 1 | 1 | 1 | 1 | 1110 | 0.5474 | 0.9149 | 123.4740 | 42.5244 | 41.3810 | 43.7315 | 974.8450 | 0.9610 | |
| 1 | 1 | 1 | 2 | 960 | 0.4598 | 0.9746 | 111.2590 | -25.8843 | 37.2086 | 158.5208 | 822.1604 | 0.9619 | |
| 1 | 1 | 1 | 3 | 615 | 0.6501 | 1.0204 | 87.0280 | 49.8521 | 32.1714 | 251.5691 | 260.6179 | 0.9762 | |
| 1 | 1 | 1 | 4 | 505 | 0.5952 | 1.0238 | 78.7320 | -87.1321 | 28.4219 | 319.5485 | 726.0277 | 0.9656 | |
| 1 | 1 | 1 | 5 | 659 | 0.2528 | 1.0285 | 89.7320 | 82.5337 | 29.5422 | 391.9727 | 236.2291 | 0.9677 | |
| 1 | 1 | 1 | 6 | 121 | 0.7141 | 0.0831 | 135.2920 | -16.7628 | 46.1163 | 431.9917 | 214.8430 | 0.1787 | |
| 1 | 1 | 1 | 7 | 536 | 0.6453 | 0.9542 | 84.0170 | 64.2546 | 30.0115 | 433.2612 | 209.7463 | 0.9487 | |
| 1 | 1 | 1 | 8 | 681 | 0.5079 | 1.0068 | 92.1960 | -11.9522 | 31.9308 | 458.3069 | 924.1924 | 0.9660 | |
| 1 | 1 | 1 | 9 | 535 | 0.5647 | 1.0327 | 80.6840 | -21.2194 | 28.8740 | 459.6486 | 951.5346 | 0.9781 | |
| 1 | 1 | 1 | 10 | 716 | 0.7126 | 0.9483 | 97.4070 | 30.6232 | 36.2126 | 481.1550 | 327.2556 | 0.9650 | |
| 1 | 1 | 1 | 11 | 581 | 0.4707 | 1.0460 | 83.5460 | 16.7058 | 29.0165 | 512.1067 | 535.4836 | 0.9798 | |
| 1 | 1 | 1 | 12 | 424 | 0.6697 | 0.9057 | 76.6980 | -0.7030 | 27.2480 | 513.3656 | 827.5660 | 0.9381 | |
| 1 | 1 | 1 | 13 | 672 | 0.3028 | 1.0515 | 89.6160 | 54.8402 | 30.0091 | 664.3988 | 910.4613 | 0.9753 | |
| 1 | 1 | 1 | 14 | 639 | 0.6925 | 0.7673 | 102.3020 | -47.1779 | 34.6134 | 696.6166 | 708.1987 | 0.9013 | |
| 1 | 1 | 1 | 15 | 446 | 0.5622 | 1.0340 | 73.6220 | -45.9194 | 26.3326 | 706.1121 | 611.8700 | 0.9867 | |
| 1 | 1 | 1 | 16 | 390 | 0.4855 | 1.0457 | 68.4610 | 56.1077 | 23.9024 | 762.4923 | 1.0013e+03 | 0.9799 | |
| 1 | 1 | 1 | 17 | 667 | 0.4228 | 1.0335 | 90.0540 | 80.7466 | 30.6970 | 800.7271 | 1.0831e+03 | 0.9723 | |
| 1 | 1 | 1 | 18 | 1270 | 0.6473 | 0.9639 | 128.6760 | -35.5204 | 46.4028 | 859.8087 | 1.1006e+03 | 0.9747 | |
| 1 | 1 | 1 | 19 | 521 | 0.3349 | 0.9181 | 84.4470 | -35.9217 | 26.8014 | 932.7927 | 109.1075 | 0.9371 | |
| 1 | 1 | 1 | 20 | 653 | 0.5134 | 1.0100 | 90.1370 | -44.0891 | 31.2663 | 946.3369 | 1.1757e+03 | 0.9746 | |
| 1 | 1 | 1 | 21 | 463 | 0.5381 | 1.0502 | 74.4320 | 66.3822 | 26.5629 | 962.7775 | 1.1431e+03 | 0.9809 | |
| 1 | 1 | 1 | 22 | 604 | 0.4701 | 0.9373 | 89.9880 | 30.5034 | 29.8651 | 977.0430 | 417.2616 | 0.9482 | |
| 1 | 1 | 1 | 23 | 1168 | 0.4655 | 0.8565 | 130.9090 | 52.5828 | 41.4510 | 992.3647 | 1.1871e+03 | 0.9374 | |
| 1 | 1 | 1 | 24 | 342 | 0.4839 | 1.0664 | 63.4820 | 71.7548 | 22.3965 | 992.7222 | 211.3099 | 0.9744 | |
| 1 | 1 | 1 | 25 | 674 | 0.2244 | 1.0476 | 89.9140 | 47.6197 | 29.8071 | 1.0570e+03 | 1.2216e+03 | 0.9868 | |
| 1 | 1 | 1 | 26 | 1086 | 0.4382 | 0.9790 | 118.0650 | -38.2795 | 39.3556 | 1.0997e+03 | 347.2145 | 0.9653 | |
| 1 | 1 | 1 | 27 | 464 | 0.6689 | 1.0090 | 76.0200 | -21.4977 | 28.4413 | 1.1703e+03 | 387.5948 | 0.9789 | |
| 1 | 1 | 1 | 28 | 797 | 0.2938 | 1.0297 | 98.6230 | 41.9659 | 32.6986 | 1.1891e+03 | 128.8946 | 0.9827 | |
| 1 | 1 | 1 | 29 | 610 | 0.4662 | 0.9865 | 88.1520 | -8.7514 | 29.7812 | 1.3397e+03 | 1.1002e+03 | 0.9713 | |
